# Antibacterial Activity and Anticancer Activity of *Rosmarinus officinalis* L. Essential Oil Compared to That of Its Main Components

**DOI:** 10.3390/molecules17032704

**Published:** 2012-03-05

**Authors:** Wei Wang, Nan Li, Meng Luo, Yuangang Zu, Thomas Efferth

**Affiliations:** 1 Key Laboratory of Forest Plant Ecology, Ministry of Education, Northeast Forestry University, Hexing Road 26, Harbin 150040, China; E-Mails: wangwping780110@163.com (W.W.); super8shen@yahoo.com.cn (N.L.); 943259819@qq.com (M.L.); 2 Engineering Research Center of Forest Bio-preparation, Ministry of Education, Northeast Forestry University, Hexing Road 26, Harbin 150040, China; 3 Department of Pharmaceutical Biology, Institute of Pharmacy and Biochemistry, University of Mainz, Mainz 55099, Germany; E-Mail: efferth@uni-mainz.de

**Keywords:** *Rosmarinus officinalis* L., 1,8-cineole, α-pinene, β-pinene, antibacterial activities, cytotoxicity

## Abstract

In this study, *Rosmarinus officinalis* L. essential oil and three of its main components 1,8-cineole (27.23%), α-pinene (19.43%) and β-pinene (6.71%) were evaluated for their *in vitro* antibacterial activities and toxicology properties. *R. officinalis* L. essential oil possessed similar antibacterial activities to α-pinene, and a little bit better than β-pinene, while 1,8-cineole possessed the lowest antibacterial activities. *R. officinalis* L. essential oil exhibited the strongest cytotoxicity towards three human cancer cells. Its inhibition concentration 50% (IC_50_) values on SK-OV-3, HO-8910 and Bel-7402 were 0.025‰, 0.076‰ and 0.13‰ (v/v), respectively. The cytotoxicity of all the test samples on SK-OV-3 was significantly stronger than on HO-8910 and Bel-7402. In general, *R. officinalis* L. essential oil showed greater activity than its components in both antibacterial and anticancer test systems, and the activities were mostly related to their concentrations.

## 1. Introduction

In the last few years, due to the misuse of antibiotics and an increasing incidence of immunodeficiency-related diseases, the development of microbial drug resistance has become more and more of a pressing problem. Recently, phytochemicals with antimicrobial potential have been extensively explored to identify components for possible medical applications [1].

Cancer is now a major cause of death in the World. Cancer is characterized by unlimited growth, invasion, and metastasis of cells, whereas benign tumors are self-limiting, non-invasive, and nonmetastasizing [2,3,4]. Synthetic drugs are often the only option for cancer chemotherapy [5,6,7]. However, most synthetic drugs kill not only tumor cells, but also normal cells, and most have severe side effects [8]. There is, therefore, an urgent need for novel treatment options with improved features. As pointed out recently, natural products from medicinal plants represent a fertile ground for the development of novel anticancer agents [9]. 

Essential oils or some of their components are used in perfumes and make-up products, in sanitary products, in dentistry, in agriculture, as food preservatives and additives, and as natural remedies [10]. In recent years, due to the new interest in natural products, plant essential oils have come more into the focus of phytomedicine [9,11]. It is important to develop a better understanding in basic research applications, especially, of the anti-microbial and anti-oxidant activities of essential oils as well as their potential anti-cancer activity [12,13].

*Rosmarinus officinalis* L. belongs to the Lamiaceae family of herbs, which in addition to being used as a food flavoring, is also known medicinally for its powerful antibacterial, antimutagenic properties, and as a chemopreventive agent [14]. *Rosmarinus officinalis* L. is also widely used today as a food preservative [15,16].

In this paper, we report the results of a study aimed at evaluating and comparing the *in vitro* antibacterial activities and toxicology properties of *R. officinalis* L. essential oil and three of its components 1,8-cineole, α-pinene and β-pinene.

## 2. Results and Discussion

### 2.1. Antibacterial Activities

Comparative antibacterial activity results of *R. officinalis* L. essential oil and three of its main components (1,8-cineole, α-pinene and β-pinene) are given in Table 1. Five microorganisms consisting of three Gram-positive bacteria and two Gram-negative bacteria were tested. 

*R. officinalis* L. essential oil possessed similar antibacterial activities to α-pinene, and a little bit better than β-pinene, while 1,8-cineole possessed lowest antibacterial activities (Table 1). For *R. officinalis* L. essential oil, α-pinene and β-pinene, the MIC values ranged from 0.0313% to 0.25% (v/v), and the MBC values ranged from 0.0625% to 0.25% (v/v), respectively. For 1,8-cineole, the MICs were from 1.25% to 5% (v/v), and the MBCs were from 2.5% to 5% (v/v), except for *Staphylococcus epidermidis’* MBC value, which was more than 5%. For *R. officinalis* L. essential oil, α-pinene and β-pinene, Gram-positive bacteria were a little bit more sensitive than Gram-negative bacteria.

**Figure 1:**
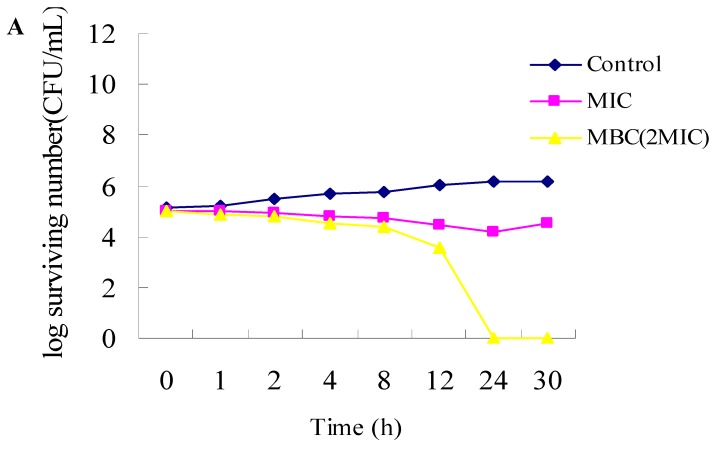

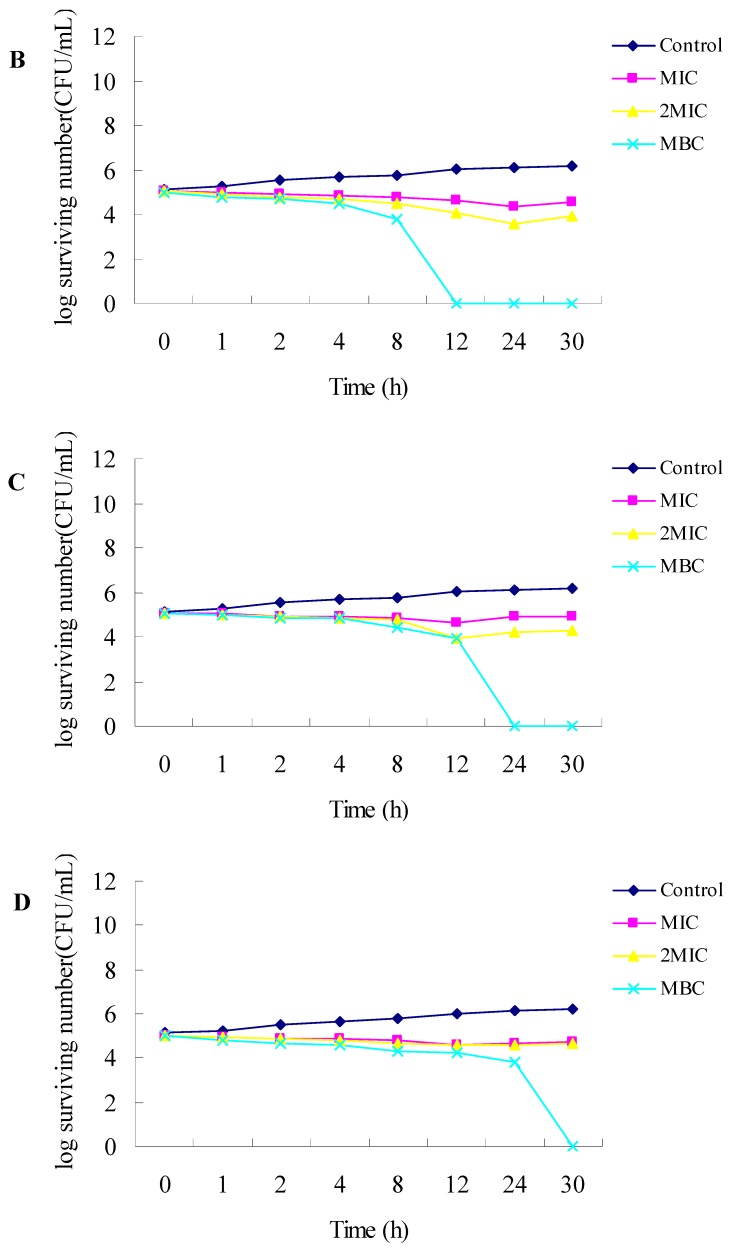
Time-kill curves of *R. officinalis* L. essential oil and three of its main components towards *Staphylococcus aureus*. (**A**) The concentrations used for *R. officinalis* L. essential oil were: 0.0313% (MIC) and 0.0625% (2MIC, MBC); (**B**) The concentrations used for α-pinene were MIC (0.0313%), 2MIC (0.0625%) and MBC (0.125%); (**C**) The concentrations used for β-pinene were MIC (0.0313%), 2MIC (0.0625%) and MBC (0.125%); (**D**) The concentrations used for 1,8-cineole were MIC (1.25%), 2MIC (2.5%) and MBC (5%). The control did not contain essential oil.

The time-kill curves of *R. officinalis* L. essential oil, α-pinene, β-pinene and 1,8-cineole towards a Gram-positive bacterium (*Staphylococcus aureus*) are shown in Figure 1. MIC had a bacteriostatic effect on the bacterial population of *Staphylococcus aureus* within the first 12 h, while MBC (2MIC) had a lethal effect on bacteria within 24 h by *R. officinalis* L. essential oil. For α-pinene, MIC and 2MIC had a bacteriostatic effect on *Staphylococcus aureus* within the first 24 h, but after that, the microorganisms treated with MIC increased faster than microorganisms treated with 2MIC; the MBC had a lethal effect on bacteria within the first 12 h, whereas the bacteria were completely killed within 24 h by β-pinene. For 1,8-cineole, MIC and 2MIC had similar bacteriostatic effects within 12 h, and the bacteria were completely killed within 30 h.

### 2.2. Cytotoxic Activity towards Cancer Cells

**Figure 2:**
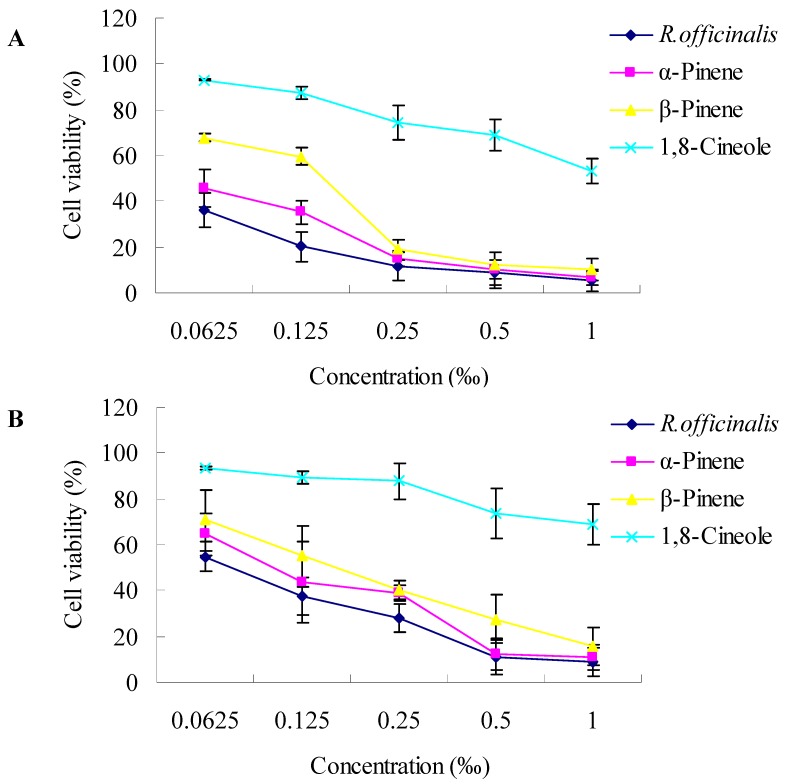

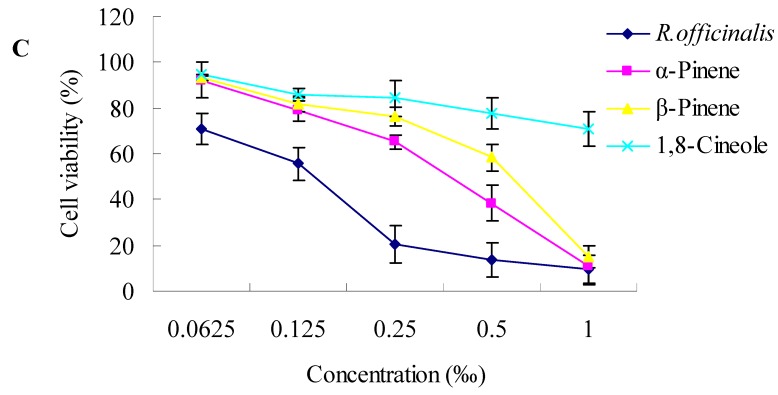
Dose-dependent cytotoxicity of *R. officinalis* L. essential oil and three of its main components (1,8-cineole, α-pinene and β-pinene; 48 h exposure) towards SK-OV-3 (**A**), HO-8910 (**B**) and Bel-7402 (**C**) cell lines as determined by the MTT assay. Values are expressed as means ± SD of three independent experiments. Standard deviations were less than 10%.

To investigate the cytotoxic activities, three human tumor cell lines, SK-OV-3, HO-8910 and Bel-7402, were exposed to increasing concentrations of *R. officinalis* L. essential oil and three of its main components (1,8-cineole, α-pinene and β-pinene). Cell viability was determined by the MTT assay. As shown in Figure 2, the essential oils revealed different cytotoxic activities towards the three human cancer cell lines investigated. In general, a dose-dependent decrease in the survival of the three tumor cell lines was observed. At a concentration of 0.0625‰ (v/v), the cell viability treated by *R. officinalis* L. essential oil, α-pinene, β-pinene and 1,8-cineole for SK-OV-3 were 36.13%, 45.85%, 67.77% and 93.03%, respectively. At a concentration of 1‰ (v/v), *R. officinalis* L. essential oil, α-pinene and β-pinene exhibited strong cytotoxicities towards the three tumor cell lines, cell viability was lower than 11%, however, cells treated with 1,8-cineole still grew well, and exhibited poor cytotoxicities. *R. officinalis* L. essential oil exhibited the strongest cytotoxicities towards all the three cancer cells than its components tested. 

The concentrations providing 50% inhibition (IC_50_) values of *R. officinalis* L. essential oil against SK-OV-3, HO-8910 and Bel-7402 were 0.025‰, 0.076‰ and 0.13‰ (v/v), respectively (Table 2). IC_50_ values for α-pinene against SK-OV-3, HO-8910 and Bel-7402 were 0.052‰, 0.11‰ and 0.32‰, respectively (v/v), whereas the IC_50_ values for β-pinene against these three cell lines were 0.12‰, 0.16‰ and 0.43‰ (v/v), respectively. However, the IC_50_ values for 1,8-cineole against these three cell lines were 1.10‰, 2.90‰ and 3.47‰ (v/v), respectively, much higher than *R. officinalis* L. essential oil, α-pinene and β-pinene. SK-OV-3 was the most sensitive cell line compared to HO-8910 and Bel-7402.

## 3. Experimental

### 3.1. Essential Oils

*Rosmarinus officinalis* L. essential oil was obtained via steam distillation, and 19 components were identified in the oil, representing 97.97% of the oil. The major constituents of the oil were 1,8-cineole (27.23%), α-pinene (19.43%), camphor (14.26%), camphene (11.52%) and β-pinene (6.71%) [16]. 1,8-Cineole, α-pinene and β-pinene were from commercial sources (Si Chuan Province, China); all the samples were stored in glass vials with Teflon sealed caps at −20 ± 0.5 °C in the absence of light. 

### 3.2. Antimicrobial Activity

#### 3.2.1. Microorganisms

*Bacillus subtilis* (Bs), *Staphylococcus aureus* (Sa), *Staphylococcus epidermidis* (Se), *Escherichia coli* (Ec) and *Pseudomonas aeruginosa* (Pa) were test strains derived from Type Culture Collection (ATCC, USA; NCTC, UK; DSM, Germany). The microorganisms were cultured overnight on agar and aerobically incubated at 37 °C.

#### 3.2.2. Determinition of MICs and MBCs Values

The MIC (minimal inhibitory concentration) and MBC (minimal bactericidal concentration) tests were performed by the broth microdilution method [17]. The essential oils were dissolved in sterilized physiological saline solution (0.9%) supplemented with Tween-80 (Sigma) at final concentration of 0.5% (v/v). Serial doubling dilutions of the essential oils were prepared in a 96-well microtiter plate ranging from 10% to 0.125%. Each dilution (100 μL) was dispensed into the wells, then inoculated with 100 μL of the bacterial suspension. The final concentration of each strain was adjusted to 10^5^–10^6^ CFU/mL. The MIC is defined as the lowest concentration of the essential oils, at which the microorganism being tested does not demonstrate visible growth. The MBC is the lowest concentration without colony growth on the agar plates, determined by seeding 10 μL from each well on a plate which was then incubated for further 24 h at 37 °C. All experiments were performed in triplicate.

#### 3.2.3. Time-Kill Dynamic Curves

Time-kill dynamic procedures were performed as described by Avila *et al.* [18] with minor modifications. The final concentration of suspension of the strain was adjusted to 10^5^–10^6^ CFU/mL. MICs, 2MICs and MBCs of *R. officinalis* L. essential oil and its main components were selected for use in the time-kill dynamic procedure. After incubating for 0, 1, 2, 4, 8, 12, 24 and 30 h with the broth micro dilution method, liquids (50 μL) were removed from the test solution for ten-fold serial dilution. Thereafter, a 50 μL liquid from each dilution was spread on the surface of the agar plates and incubated at 37 °C for 24 h, and the number of CFU/mL was counted. The solution with no essential oil was used as a control. Time-kill curves were constructed by plotting the log number of CFU/mL against time (h).

### 3.3. Cytotoxicity Assay

#### 3.3.1. Maintenance of Human Cancer Cell Lines

Human ovarian cancer cell lines (SK-OV-3 and HO-8910) and human hepatocellular liver carcinoma cell line (Bel-7402) were purchased from China Center for Type Culture Collection (Wuhan, China). These cell lines were grown and maintained in a humidified incubator at 37 °C with a 5% CO_2_ atmosphere. Dulbecco’s modified Eagle’s medium (DMEM) supplemented with 10% fetal bovine serum (FBS), 100 U/mL penicillin and 100 μg/mL streptomycin was used for the cell cultures.

#### 3.3.2. Cytotoxicity Assay

The cytotoxic effects of the *R. officinalis* L. essential oil and three of its main components (1,8-cineole, α-pinene and β-pinene) on three human tumor cell lines were assayed by the MTT assay [19]. The cells were seeded at a density of 5 × 10^4^ cells/well. The essential oils were serially double diluted from 1‰ to 0.0625‰ (v/v), and 200 μL liquid of each concentration was applied to the wells of a 96-well plate containing confluent cell monolayers (six wells per concentration). The dilution medium without the sample served as a control. After 48 h of incubation, MTT solution (5 mg/mL) was then added to each well, and the formazan precipitate was dissolved in 200 μL dimethyl sulfoxide after 4 h incubation. The content of the wells was homogenized on a microplate shaker for 5 min. The optical densities (OD) were measured on a microplate ELISA reader at 492 nm. All tests and analyses were run in triplicate and mean values were recorded. The cell survival curves were calculated after comparing with the control. The percentage viability was calculated as follows:

$$\%\mathrm{viability}=\frac{\mathrm{mean}\;\mathrm{absorbance}\;\mathrm{of}\;\mathrm{treated}\;\mathrm{wells}}{\mathrm{mean}\;\mathrm{absorbance}\;\mathrm{of}\;\mathrm{untreated}\;\mathrm{wells}\;(\mathrm{no}\;\mathrm{oil})}\times 100$$

## 4. Conclusions

In recent decades, essential oils have been in increasing demand by manufacturers of foods, cosmetics and pharmaceuticals, hence the importance of conducing studies on essential oils, lies not only in their chemical characterization, but also in the possibility of linking the chemical contents with particular functional properties. In this regard, it is advisable to use methods for the assessment of biological activities that not only highlight aromatic or preservative activities but also correlate with functional properties that might be potentially useful for pharmaceuticals, nutraceuticals and cosmetic applications. Following this idea, we assessed the comparative antibacterial and anticancer activities of *Rosmarinus officinalis* L. essential oil and three of its main components 1,8-cineole, α-pinene and β-pinene. *R. officinalis* L. essential oil exhibited the strongest antibacterial and cytotoxic activities towards SK-OV-3, HO-8910 and Bel-7402 human tumor cell lines, which were in order: *Rosmarinus officinalis* L. essntial oil > α-pinene > β-pinene > 1,8-cineole.

It is very difficult to attribute the biological activities of a total essential oil to one or a few active principles, because an essential oil always contains a mixture of different chemical compounds. In addition to the major compounds, also minor compounds may make a significant contribution to the oil’s activity [16]. From the results above we could infer that the antibacterial and anticancer activities of *Rosmarinus officinalis* L. essntial oil is the cooperative results of their components.

## Figures and Tables

**Table 1:** Minimal inhibitory concentrations (MICs, % v/v) and minimal bactericidal concentrations (MBCs, % v/v) of *Rosmarimus officinalis* L. essential oil and three of its main components.

	*R.officinalis* L.		α-Pinene		β-Pinene		1,8-Cineole		Streptomycin (mg/mL)	
	MIC	MBC	MIC	MBC	MIC	MBC	MIC	MBC	MIC	MBC
Bs	0.0625	0.0625	0.0625	0.0625	0.0625	0.0625	1.25	2.5	0.0313	0.125
Sa	0.0313	0.0625	0.0313	0.125	0.0313	0.125	1.25	5	0.0313	>0.125
Se	0.0313	0.0625	0.0313	0.0625	0.0625	0.125	5	>5	0.00781	0.125
Ec	0.0625	0.125	0.0625	0.125	0.0625	0.125	1.25	5	0.0625	>0.125
Pa	0.0625	0.25	0.0625	0.25	0.0625	0.25	2.5	2.5	0.125	>0.125

**Table 2:** 50% Inhibition concentrations (IC_50_, ‰ v/v) values for *Rosmarimus officinalis* L. essential oil and three of its main components toward SK-OV-3, HO-8910 and Bel-7402 as determined by the MTT assay.

	*R. officinalis* L.	α-Pinene	β-Pinene	1,8-Cineole
SK-OV-3	0.025	0.052	0.12	1.10
HO-8910	0.076	0.11	0.16	2.90
Bel-7402	0.13	0.32	0.43	3.47
